# Gut microbial features and dietary fiber intake predict gut microbiota response to resistant starch supplementation

**DOI:** 10.1080/19490976.2024.2367301

**Published:** 2024-06-24

**Authors:** Sri Lakshmi Sravani Devarakonda, Dorothy K. Superdock, Jennifer Ren, Lynn M. Johnson, Aura (Alex) P. Loinard-González, Angela C. Poole

**Affiliations:** a Division of Nutritional Sciences, Cornell University, Ithaca, NY, USA; b Cornell Statistical Consulting Unit, Cornell University, Ithaca, NY, USA

**Keywords:** resistant starch, gut microbiome, gut microbiota, short-chain fatty acid, precision nutrition, interindividual, *AMY1*, amylase, dietary fiber

## Abstract

Resistant starch (RS) consumption can have beneficial effects on metabolic health, but the response, in terms of effects on the gut microbiota and host physiology, varies between individuals. Factors predicting the response to RS are not yet established and would be useful for developing precision nutrition approaches that maximize the benefits of dietary fiber intake. We sought to identify predictors of gut microbiota response to RS supplementation. We enrolled 76 healthy adults into a 7-week crossover study with 59 individuals completing the study. Participants consumed RS type 2 (RS2), RS type 4 (RS4), and digestible starch, for 10 d each with 5-d washout periods in between. We collected fecal and saliva samples and food records during each treatment period. We performed 16S rRNA gene sequencing and measured fecal short-chain fatty acids (SCFAs), salivary amylase (*AMY1*) gene copy number, and salivary amylase activity (SAA). Dietary fiber intake was predictive of the relative abundance of several amplicon sequence variants (ASVs) at the end of both RS treatments. *AMY1*-related metrics were not predictive of response to RS. SAA was only predictive of the relative abundance of one ASV after digestible starch supplementation. Interestingly, SCFA concentrations increased the most during digestible starch supplementation. Treatment order (the order of consumption of RS2 and RS4), alpha diversity, and a subset of ASVs were predictive of SCFA changes after RS supplementation. Based on our findings, dietary fiber intake and gut microbiome composition would be informative if assessed prior to recommending RS supplementation because these data can be used to predict changes in specific ASVs and fecal SCFA concentrations. These findings lay a foundation to support the premise that using a precision nutrition approach to optimize the benefits of dietary fibers such as RS could be an effective strategy to compensate for the low consumption of dietary fiber nationwide.

## Introduction

Resistant starch (RS), a form of dietary fiber, resists degradation by human enzymes and reaches the large intestine where it can be fermented by colonic microbes to produce beneficial metabolites.^1^ RS is classified into five subtypes (RS1–RS5), and most human studies use RS types 2, 3, and 4. RS2 refers to starch with densely organized granules, which is believed to hinder effective attachment of digestive enzymes. RS3 is created through the process of retrogradation of gelatinized starch resulting in a crystalline structure that is inaccessible to digestive enzymes. RS4 is created through chemical modification of starch by processes such as dextrinization and esterification, rendering it resistant to digestion by human enzymes.^2^ Different microbes can selectively ferment different types of RS to produce short-chain fatty acids (SCFAs).^3^ SCFAs play a role in important metabolic processes including appetite regulation, glucose homeostasis, and lipid metabolism.^1,4–6^

Interestingly, several groups have reported interindividual variability in gut microbiota response and host physiological response (e.g. improvements in glucose metabolism) to supplementation with dietary fiber, including RS2 and RS4.^7–14^ Host and microbial factors prior to RS supplementation may be predictive of, and responsible for, the interindividual variability in gut microbiota response to RS. Previous studies have shown that habitual dietary fiber intake^2,15^ and baseline microbiome composition^16^ affect gut microbiota response to several nonstarch polysaccharides, another major category of dietary fiber. Also, host dietary practices can alter microbial diversity, which in turn can affect response to dietary interventions.^17^

In addition, the genotype of the host may influence microbial response. A proposed genetic factor that may predict variable response to RS is *AMY1*, a gene copy number (CN) variant, which encodes the salivary amylase enzyme.^11,18^ *AMY1* CN is correlated with salivary amylase activity (SAA), which helps to initiate starch digestion. *AMY1* CN has been associated with BMI, glucose metabolism, and gut microbiome composition.^18–23^ Thus, we posited that *AMY1* CN or SAA may be predictive of response to dietary fiber intake.

Although preintervention differences in host and microbial characteristics are thought to be responsible for the reported variability, as of yet, the field has not established predictors of gut microbiota or host physiological response to RS supplementation. This knowledge would aid in developing precision nutrition approaches and, in particular, tailoring dietary fiber recommendations to individuals seeking health benefits from RS consumption.

Here, we address a gap in knowledge regarding the factors underlying interindividual variation in gut microbiota response to RS supplementation. In this dietary intervention study, we supplemented participants’ diets with two types of RS, RS2 and RS4, as well as a control digestible corn starch to assess the resulting changes in gut microbiota composition and SCFA concentrations. Then we evaluated the ability of the candidate predictors—*AMY1* CN, SAA, prior gut microbiome composition, and dietary fiber intake—to explain interindividual differences in the response to each treatment. Further, because we used a crossover design, we were able to evaluate the effect of the order of treatments on the observed results.

## Materials and methods

### Study participants

This study was conducted at Cornell University in Ithaca, New York, between October and December 2020. Participants were recruited via advertisements using flyers and e-mail listservs in and around the Cornell University campus. All human-related procedures and sample and data collection were approved by the Cornell University Institutional Review Board for Human Participant Research (Protocol Number: 1902008575) prior to recruitment and enrollment of participants. Study participants included healthy males and healthy, non-pregnant or lactating females 18–59 years old. Baseline participant characteristics can be found in Table 1. Exclusion criteria included a history of gastrointestinal diseases or surgeries; type 1 or type 2 diabetes, prediabetes or impaired glucose tolerance, self-reported untreated thyroid condition; use of antibiotics 6 months prior to the start of the study; and chronic alcohol intake (>5 drinks/day). The full list of inclusion and exclusion criteria is provided in Table S1. This trial was registered at clinicaltrials.gov as NCT05743790 on February 24, 2023.

**Table 1.** Baseline characteristics for all participants assigned to a study arm.

	Group A (*n* = 35)	Group B (*n* = 33)	Total	p value
Age in years	28 ± 9	27 ± 11	27 ± 10	0.376
Sex, n (%)				0.542
Female	22 (62.9%)	24 (72.7%)	46 (67.6%)	
Male	13 (37.1%)	9 (27.3%)	22 (32.4%)	
Body fat percentage				
Female	26.2 ± 7.9	24.9 ± 6.6	25.50 ± 7.2	0.531
Male	18.0 ± 5.1	20.40 ± 7.0	19.00 ± 5.9	0.351
Energy-adjusted dietary fiber intake at baseline in grams	12.7 ± 5.5	11.0 ± 4.3	11.9 ± 5.0	0.235

There were 35 participants in Group A and 33 in Group B, for a total of 68 who were assigned to a study arm. Data are presented as mean (standard deviation) or n (%). There were no significant differences between Group A and B at baseline for age, sex, body fat percentage for males, body fat percentage for females, and energy-adjusted dietary fiber intake at baseline. T-tests and Wilcoxon rank sum tests were used for numerical variables and chi-squared tests were used for binary variables.

### Study design

**Figure 1.**
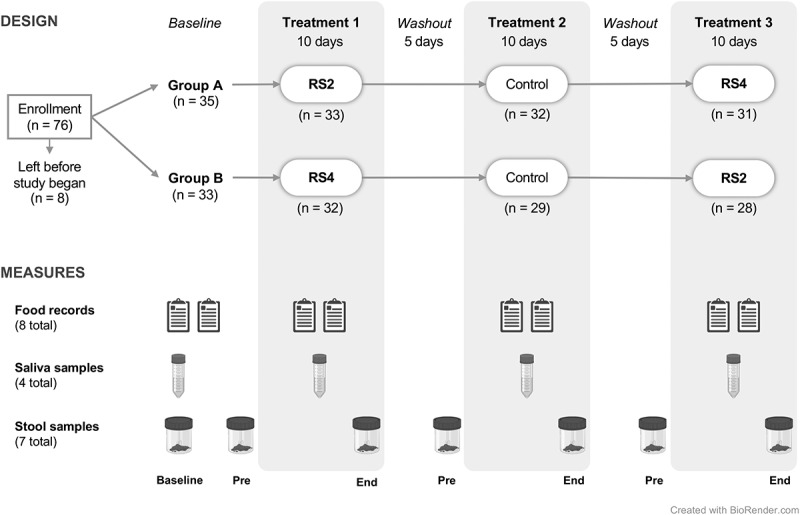
Study design.

We enrolled eligible participants into a 7-week open-label crossover dietary intervention study (CONSORT diagram: Figure S1). Each participant was assigned a study identification (ID) number based on the order in which they were enrolled in the study. All participants with an odd ID number were allocated to Group A, and all participants with an even ID number were allocated to Group B, achieving a 1:1 balanced allocation. A single participant with an odd ID number was allocated to Group B to balance the groups. Participants were instructed to consume crackers containing RS2, RS4, or a digestible control starch (Ctl), in their assigned treatment order, each for 10 d (Figure 1), in addition to their normal dietary intake. The groups received treatments in the following order. Group A: Treatment 1 = RS2, Treatment 2 = Ctl, Treatment 3 = RS4; Group B: Treatment 1 = RS4, Treatment 2 = Ctl, Treatment 3 = RS2. The three treatment periods were separated by 5-d washout periods when no study crackers were consumed. Participants collected fecal samples prior to (“PreRS2,” “PreCtl,” or “PreRS4”) and at the end of (“EndRS2,” “EndCtl,” or “EndRS4”) the treatment periods (Figure 1: see “Pre” and “End” time points).

Participants were enrolled in one of the two arms (Groups A and B) of a 7-week crossover dietary intervention study during which we supplemented their diets with crackers containing one of the three starches: RS2 = resistant starch type 2, RS4 = resistant starch type 4, Control = digestible starch. Each treatment lasted for 10 d, and there were 5-d washouts between treatments. Stool samples were collected at baseline and at the beginning (Pre) and end (End) of each treatment. Please see CONSORT flow diagram (Figure S1). This figure was created using BioRender.

### Dietary supplementation

Participants were provided with crackers that contained RS2 (HI-MAIZE® 260 starch, Ingredion), RS4 (VERSAFIBE™ 1490 starch, Ingredion), or a digestible control starch (AMIOCA™ TF starch, Ingredion). Study cracker formulations are listed in Table S2a. During Treatments 1 and 3, we aimed to provide 30 g of each type of RS in a daily serving of 120 g of crackers for 7 d after a 3-d ramp-up period to minimize gastrointestinal discomfort. The daily serving size for the control cracker after the ramp-up period of 3 d was also 120 g. Thus, during each 10-d treatment period, we followed a dose escalation of study crackers where participants gradually increased the dosage over 3 d to reach a final dose of 120 g of crackers per day for the last 7 d (Day 1: 25%, Day 2: 50%, Day 3: 75%, Day 4–Day 10: 100%). The RS2, RS4, and control crackers were approximately matched for total carbohydrate, fat, and protein content, but the control cracker contained more calories than the RS crackers (Table S2b). Following production, the crackers were analyzed for RS and total dietary fiber content by Medallion Laboratories (Minneapolis, MN) using AOAC 2002.02 and AOAC 991.43 methods, respectively (Table S3). The method AOAC 2002.02 accurately quantifies RS2 but not RS4, so it is not providing an accurate measurement of RS in the RS4 crackers.^24,25^ However, the method AOAC 991.43 does detect RS4, so by deduction, the AOAC 991.43 measurement is indicating the approximate amount of RS4 (less the 1.92 g of total dietary fiber from the other ingredients, which are also in the control cracker).^26^ After baking, the amount of RS in RS2 crackers was reduced from 30 g to 21.27 g per serving, possibly due to starch gelatinization, and this may have also occurred in the RS4 crackers. Participants were asked to maintain their habitual dietary intake and physical activity across interventions and to avoid taking prebiotic- or probiotic-added foods, drinks, or supplements throughout the study. Study crackers were provided in individual bags containing a preportioned daily supply by weight. At the end of each treatment period, participants were asked to complete a short questionnaire to indicate the percentage of crackers consumed per day as an indicator of adherence to the protocol. Data from participants who reported a low adherence to cracker consumption were excluded from analysis. Low adherence was defined as having consumed <75% of study crackers for 5 or more days during the treatment or <50% on the day of or day before collecting their End time point fecal sample.

### Anthropometric measurements

At baseline, we measured the participants’ total body fat percentage with a Tanita SC-240 Total Body Composition Analyzer using Bioelectrical Impedance Analysis (BIA) as per the manufacturer’s instructions.

### Dietary intake data

During the baseline and treatment periods, we instructed participants to complete two nonconsecutive 1-d food records of one weekday and one weekend day, excluding study cracker consumption, using the Automated Self-Administered 24-hour (ASA24) Dietary Assessment Tool 2020 developed by the National Cancer Institute, Bethesda, MD (https://epi.grants.cancer.gov/asa24.).^27^ Data were averaged from the two food records for each participant within the baseline week and each of the three treatments (Figure S2a). Data were excluded if only one of the two records was completed.

### Saliva collection

We obtained 5 ml of saliva at baseline and during each treatment for a total of four saliva samples throughout the study for each participant (Figure 1). Participants were instructed to refrain from brushing their teeth for a minimum of 6 hours and to avoid consuming any food or beverages including water for a minimum of 30 minutes prior to sample collection. They were then instructed to accumulate saliva in their mouth and express it into a 50 ml sterile conical tube. Saliva samples were stored on ice immediately after collection, aliquoted within 3 hours, and stored at −80°C.

### AMY1 copy number determination by qPCR and ddPCR

Genomic DNA was extracted from saliva samples using the QIAamp 96 DNA Blood Kit, QIAamp Blood Mini Kit, and the QIAamp Investigator Kit (Qiagen, cat # 51161, 51104, 56504). We performed qPCR using primers, previously described in Poole et al.,^18^ to amplify *AMY1* paralogs and our reference gene, *EIF2B2* (CN = 2). For each gene, each qPCR reaction consisted of 1 μl genomic DNA normalized to 5 ng/μl, 0.5 μl of each forward and reverse primer at 10 μM, 3 μl of PCR grade H2O, and 5 μl iTaq™ Universal SYBR® Green Supermix (BioRad, cat # 1725122), for a total volume of 10 μl per reaction. The qPCR conditions were as follows: initial denaturation at 95°C for 5 minutes and 40 cycles of 95°C for 10 seconds and 60°C for 30 seconds on a Roche LightCycler 480 Real-Time PCR Instrument. We made a standard curve using genomic DNA NA12286 (Coriell Institute; *AMY1* CN = 2). The following genomic DNAs were used as positive controls on all qPCR plates, as their *AMY1* copy numbers have previously been estimated: NA18972, NA12873, NA10472, NA12890, NA10852, NA12043, NA11992, NA12414, NA12340, NA06994, NA12342, NA12286, NA18522, and NA19138 (Coriell Institute). All reactions including standards and blanks were performed in quadruplicate, and results were averaged for technical replicates with a coefficient of variation <0.05. At least two qPCR runs were performed for all participants, and we calculated the median value of all qPCR results to determine the final qPCR *AMY1* CN value. For digital PCR, genomic DNA was digested with the restriction enzyme *Hae*III (New England Biolabs, cat # R0108S) and diluted to a final concentration of approximately 15 ng/µl. Digital PCR was performed using Life Technologies TaqMan Copy Number Assay Id Hs07226361_cn for the *AMY1* locus and TaqMan Copy Number Reference Assay Hs06006763_cn for the AP3B1 gene to normalize for total DNA. The reactions were run on a QX100 Droplet Digital PCR System in duplicate. We averaged the median of all qPCR results and the two digital PCR results to determine the final *AMY1* CN used in our analyses (Figure S2b).

*AMY1* CN group was defined as that used in a previous publication that inspired this work^18^ because even though categorization is often used, currently, there is no consensus in the field regarding the threshold for high and low categories.^28,29^ Perry et al. reported that low starch consuming populations have a median *AMY1* CN of 5 and high starch consumers have a median of 7.^30^ Although the threshold for the effects of this gene on physiology or health is currently unknown, Perry et al.’s conclusion that positive selection occurred at this locus due to dietary adaptation suggests that the low end of phenotypic effect is CN of 5. In our cohort, salivary amylase activity (SAA) is significantly different between our *AMY1* CN groups with the mean of the high group (SAA = 145 U/ml) being over twice that of the low group (SAA = 66 U/ml) over time (*p* = 4.85 × 10^−4^).

### Salivary amylase activity assay

We measured SAA for each saliva sample in triplicate, from up to four saliva samples donated from each participant throughout the duration of the study, using the Salimetrics Salivary Alpha-Amylase Enzymatic Kit (SALIMETRICS, cat # 1–1902). The manufacturer’s protocol was followed except for the use of 300 µl amylase substrate per reaction instead of 320 µl. We averaged SAA determined for each participant to obtain the mean SAA used in our analyses (Figure S2c).

### Fecal sample collection

Participants collected fecal samples at seven time points throughout the study (Figure 1). Samples were collected from a single bowel movement and stored at −80°C within 24 hours of collection. We lyophilized an aliquot from each sample for 16S rRNA gene sequencing. Samples from study participants who reported collection of fecal samples ≥2 d after completion of the treatment were excluded from analysis.

### 16S rRNA gene sequencing

DNA extraction was performed on 0.030–0.045 g lyophilized fecal samples using the DNeasy PowerSoil 96 HTP Kit (Qiagen, cat # 12888–100), following the manufacturer’s instructions with the specifications: samples were loaded into PowerBead Plates and stored at −20°C in Bead Solution until extraction, and instead of vortexing to mechanically lyse samples, samples were placed in a BioSpec 1001 Mini-Beadbeater-96 for 3 minutes. We amplified the V4 region of the 16S rRNA gene using the universal primers 515F and barcoded 806 R^31^ and approximately 100 ng of genomic DNA from each sample in duplicate PCR reactions using 25 µl Classic++™ Hot Start Taq DNA Polymerase Master Mix (Tonbo Biosciences, cat # TB-31-5011–1000 R), 22 µl PCR water, 0.5 µl of each 10 nM primer, and 2 µl DNA. We used the PCR program previously described but with 25 cycles of amplification.^31^ We purified amplicons using Mag-Bind TotalPure (Omega Bio-tek, cat # M1378–01) using a 1.8X bead ratio, pooled 100 ng of amplicons from each sample, and performed 2 × 250 bp sequencing on an Illumina MiSeq instrument. We performed microbiome bioinformatics with QIIME 2.^32^ We demultiplexed and quality filtered raw sequence data via q2-demux. Then we resolved ASVs using DADA2^33^ via q2-dada2. We aligned ASVs with mafft^34^ via q2-alignment and constructed a phylogenetic tree using fasttree^35^ via q2-phylogeny. We used q2-diversity to calculate alpha-diversity (Faith’s Phylogenetic Diversity, Faith’s PD)^36^ after samples were subsampled without replacement to 23,081 sequences per sample based on the sample with the lowest sequence count. These rarefied data were used exclusively for calculating Faith’s PD. For differential abundance analysis, we utilized the non-rarefied feature table to retain the full complexity of the microbial communities. We used the q2-feature-classifier^37^ classify-sklearn naive Bayes classifier against the Greengenes 13_8 99% OTUs reference sequences^38^ to assign taxonomy to ASVs. We identified 6,547 ASVs within our entire dataset (all time points from 59 participants). After we calculated the mean number of ASVs present (number of ASVs with a non-zero read count) in each individual across all time points, we observed that the mean of the means was 159 ASVs per person, the median of the means was 156 ASVs per person, and the standard deviation of the means was 45.

### Short-chain fatty acid measurements

We measured SCFA concentrations in stool samples collected at baseline and at the time points Pre and End of each treatment. SCFAs including acetate, propionate, isobutyrate, butyrate, isovalerate, valeric acid, isocaproic acid, caproic acid, and heptanoic acid were quantified using ultra-performance liquid chromatography (Acquity UPLC system, Waters Corporation, Milford, MA) at the PennCHOP Microbiome Program Microbial Culture & Metabolomics Core. Total SCFA concentration was calculated as the sum of all nine SCFAs quantified.

### Statistical analysis

We performed all statistical analyses using RStudio version 4.2.1.^39^ We adjusted dietary fiber intake by energy (g/1,000 kcal/day) to account for differences in participants’ overall energy intake. We considered p-values <0.05 to be statistically significant. When adjusting p-values we used the Benjamini–Hochberg false discovery rate correction and considered a q-value (adjusted p-value) of <0.05 to be statistically significant. To calculate fold changes of ASVs, we added a pseudocount of 1 to all ASV counts prior to calculating relative abundances to avoid division by zero. Since the limit of detection of the quantification method for SCFAs was 5.0 µmol/g of stool, a count of 2.5 µmol/g was added to all SCFA measurements in the linear regression and LASSO analyses, in which logarithm or fold change was calculated, to avoid an undefined term (logarithm of zero or division by zero). We used R package ComplexHeatmap^40^ and circlize^41^ to generate all heat maps.

To assess the response of ASVs to each treatment, we used the R package MaAsLin2 (Microbiome Multivariable Association with Linear Models 2) because this software allows us to include repeat measures in longitudinal sampling (Pre and End) as well as covariates, takes into consideration the nature of ASV data (zero-inflated, high dimensional, non-normal distribution) and controls for the false discovery rate.^42^ Data from all participants were included, regardless of the order of treatment (Group). For each treatment (RS2, RS4, and Ctl), we used the min_prevalence default = 0.1 parameter to filter out the ASVs present in less than 10% of the samples collected. For example, ASVs had to be present in at least 10% of the combined samples from PreRS2 and EndRS2 to be included in the RS2 linear mixed model, leaving a total of 325 ASVs. After prevalence filtering, there were 330 ASVs included in the linear mixed model for RS4 and 365 ASVs for Ctl. Prior to fitting the models, the ASV data were normalized using total sum scaling. We used the log transformation setting within MaAsLin2’s transform parameter. For each treatment, we fit three types of models. First, we fit main effects models using time point, abbreviated TP (PreRS2 versus EndRS2, PreRS4 versus EndRS4, or PreCtl versus EndCtl), as a fixed effect and participant as a random effect in each model to identify ASVs that, on average, changed in relative abundance from Pre to End of each treatment period.

(1) $$\mathrm{Relative}\;\;\mathrm{abundance}\;\;\;\text{\textbackslash{}\textasciitilde{}}\;\;\;\mathrm{TP}\;\left\{\mathrm{Pre},\;\mathrm{End}\right\}\;+\;1|\mathrm{Participant}$$

Our next goal was to identify variables that affected the change in relative abundances of ASVs during each treatment. Thus, we added interaction terms to each of these separate models (*AMY1* CN × time point, *AMY1* CN group × time point, mean SAA × time point, baseline dietary fiber intake × time point, dietary fiber intake during treatment × time point, and treatment order × time point). Because this was a screening step, we followed up on all interactions with q < 0.25. Next, we fit main effects models using End time points only, with each candidate predictor as an independent variable in a separate model, to detect whether the candidate predicted the relative abundance of any ASVs at the end of the treatment with q < 0.05.

To determine the effect of each treatment on SCFA concentrations, we fit linear mixed models with log SCFA concentration as the response variable, time point (TP; Pre vs End) as a fixed effect, and participant as a random effect.

(2) $$\mathrm{Log}\;\left(\mathrm{SCFA}\;+\;2.5\right)\;\;\;\text{\textbackslash{}\textasciitilde{}}\;\;\;\mathrm{TP}\left\{\mathrm{Pre},\;\mathrm{End}\right\}\;+\;1|\mathrm{Participant}$$

We fit separate models for each treatment (RS2, RS4, Ctl) and SCFA (acetate, butyrate, propionate, and total).

To determine whether treatment order (Group A versus B) affected changes in SCFA concentrations from Pre to End (time point), we used linear regression models with each SCFA as the response variable and included an interaction term between Group and time point. A random effect term was included for participant. Fixed effects were tested using F tests with the Kenward-Roger approximation for the degrees of freedom, and post-hoc pairwise comparisons were performed using Tukey’s HSD method.

(3) $$\mathrm{Log}\;\left(\mathrm{SCFA}\;+\;2.5\right)\;\;\;\text{\textbackslash{}\textasciitilde{}}\;\;\;\mathrm{TP}\left\{\mathrm{Pre},\;\mathrm{End}\right\}\;x\;\;\mathrm{Group}\left\{A,\;B\right\}\;+\;\mathrm{TP}\left\{\mathrm{Pre},\;\mathrm{End}\right\}\;+\;\mathrm{Group}\left\{A,\;B\right\}\;+\;1|\mathrm{Participant}$$

To assess whether alpha diversity could predict changes in SCFA concentrations, we fit logistic regression models with SCFA change score as the response variable and Faith’s phylogenetic diversity (PD) as a fixed effect. We defined any increase in SCFA concentration from Pre to End (End – Pre > 0) to have a change score of 1, while no change or a decrease in SCFA concentration (End – Pre ≤ 0) was assigned a change score of 0.

(4) $$\mathrm{SCFA}\;\mathrm{change}\;\mathrm{score}\;\;\;\text{\textbackslash{}\textasciitilde{}}\;\;\;\mathrm{Fait}h^{\prime }s\;\mathrm{PD}\;\left\{\mathrm{Pre}\right\}$$

Because our goal is to predict the SCFA change score, we used Faith’s PD at the beginning of the treatment (Pre) as the predictor in each model. We fit separate models for each treatment (RS2, RS4, Ctl).

We used the R package glmnet^43^ to perform 10-fold cross-validated Least Absolute Shrinkage and Selection Operator (LASSO) linear regressions to model the log fold change of acetate, propionate, and butyrate concentrations between Pre and End of each treatment period (log2 [End/Pre]). For variable selection, we included the predictors: 10% prevalence-filtered ASVs at Pre, body fat percentage, sex, treatment order, *AMY1* CN, *AMY1* CN group, mean SAA, and self-reported physical activity (minutes/week of vigorous, moderate, and low-intensity activity) at baseline. We used a separate model for each SCFA and each treatment, and after 10% prevalence filtering for each treatment, there were 332 ASVs in the RS2 models, 339 ASVs in the RS4 models, and 349 ASVs in the Ctl models. To create the figure depicting the LASSO coefficients, we standardized the regression coefficients and excluded the predictor group, or treatment order, to show all the coefficients on a comparable scale. Standardization was performed by multiplying each β coefficient by the standard deviation of the relative abundance of the corresponding ASV at Pre.

## Results

### Baseline characteristics of study participants

We enrolled 76 individuals into a 7-week open-label crossover dietary intervention study with 59 individuals completing the full study (Figure 1). Study enrollment and the number of participants completing each treatment are detailed in Figure 1 (CONSORT diagram; Figure S1). We describe baseline characteristics for participants included in our analyses in Table 1. When comparing the two different treatment order groups, Group A and Group B, at baseline, there were no significant differences in age, sex, dietary fiber intake, body fat percent in males, or body fat percent in females (*p* > 0.05 for all). Of note, the average amount of dietary fiber consumed by all participants during the baseline period (11.88 g, SD = 4.98 g) was lower than the Institute of Medicine’s recommendations of 25–38 g/day depending on sex and age,^44^ and below the dietary reference intake.^45^

### RS, but not digestible starch, elicits an overall change in gut microbiome composition

**Figure 2.**
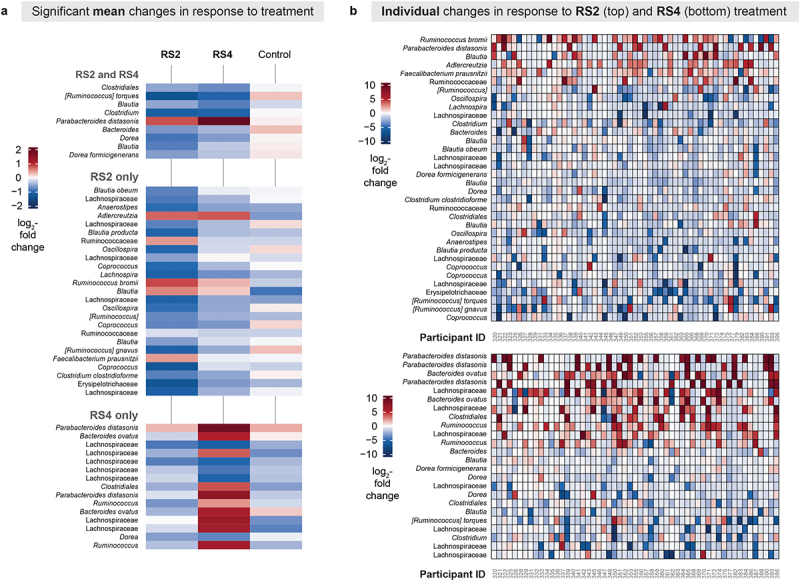
Change in gut microbiome composition after RS2 and RS4 treatments varies between individuals.

For each treatment, we used MaAsLin2 models to assess overall microbial response, which was represented by ASVs that changed in relative abundance in the study cohort from the beginning to the end of the treatment period. MaAsLin2 allows the user to create linear mixed models that include repeated measures of all the ASVs in a longitudinal dataset. Thus, we included both Pre and End measurements in each model and a random effects term for participant. All data from all participants were included, regardless of order of treatment (Group). We used a separate linear model for each treatment (RS2, RS4, and control). We observed that 34 out of 325 ASVs were differentially abundant between PreRS2 (before RS2) and EndRS2 (the end of RS2) treatment, 24 out of 330 ASVs were differentially abundant between PreRS4 and EndRS4, and none of the 365 ASVs were significantly different between PreCtl (before control) and EndCtl (end of control) treatment (q < 0.05; Figure 2; Table S4). Of the 34 ASVs identified for RS2 and the 24 ASVs identified for RS4, we found 9 ASVs shared between the two treatments. The mean log_2_ fold changes of relative abundances of the significantly differentially abundant ASVs are shown in Figure 2(a). Although we observed these population level shifts, we detected noticeable variation between individuals for RS2 and RS4 (Figure 2(b)). Measuring the degree of interindividual variability, we found that an ASV for *Ruminococcus bromii* (var = 11.09) and an ASV for *Parabacteroides distasonis* (var = 17.37) had the greatest variance in log_2_ fold change between the beginning and end of treatment with RS2 and RS4, respectively. Thus, even bacteria routinely reported as responding to RS supplementation can exhibit interindividual variability in changes in relative abundance.^7,9,46,47^ The interindividual variability in gut microbiome response may also contribute to the conflicting results reported regarding improvements in cardiometabolic health in RS intervention studies.^48–50^

(a) This heat map shows the change in mean relative abundance of ASVs across all participants by treatment (RS2, RS4, and control) for taxa that significantly differed in abundance from Pre to End of the RS2 and RS4 treatments in our MaAsLin2 models (q < 0.05). Descriptions above the taxa names on the left indicate whether the ASVs were significant for both RS2 and RS4, RS2 only, or RS4 only. Although the mean log_2_ fold change in relative abundance of taxa during the control treatment is shown, no ASVs significantly changed in response to the control treatment at q < 0.05. For each cell, colors indicate the log_2_ fold change in relative abundance of taxa between Pre and End treatment time points. Repeated taxa indicate the same taxonomic assignment to different ASVs. Data from all participants was included regardless of order of treatment. (b) These are the same differentially abundant ASVs in panel (a), but shown here are the log_2_ fold changes of the relative abundances of the ASVs for individual participants during RS2 (top) and RS4 (bottom) (RS2: *n* = 57, RS4: *n* = 55, control: *n* = 55).

### Dietary fiber intake and mean SAA are predictive of differential abundance of ASVs at the end of treatment periods

We used MaAsLin2 models to assess the ability of candidate predictors to predict differences in relative abundances between the Pre and End time points of the treatment periods. Our candidate predictors were as follows: *AMY1* CN, *AMY1* CN group (high: ≥9 copies versus low: ≤5 copies),^18^ mean SAA, dietary fiber intake during baseline, dietary fiber intake during treatment (excluding study cracker consumption), and treatment order (whether participants consumed RS2 or RS4 initially). The distribution of the candidate predictors can be found in Figure S2. Each candidate predictor, except *AMY1* CN, had an interaction with time point for at least one ASV in at least one of the treatment periods at q < 0.25 (Table S5). We used a generous threshold of q < 0.25 here as this was a screening step to identify candidates for further testing.

**Figure 3.**
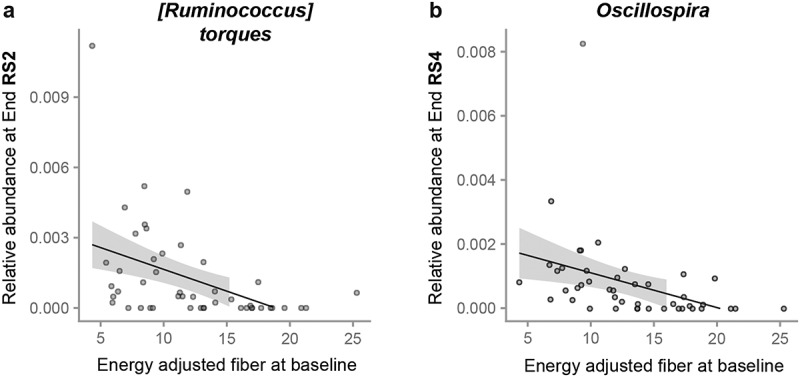
Dietary fiber intake at baseline predicts ASV relative abundances at the End of treatment.

In the next step, we fit main effects models using End time points only, with each candidate predictor as an independent variable in a separate model, to detect whether the candidate predicted the relative abundance of any ASVs at the end of the treatment. For this step of the analysis, we used a more conservative threshold of q < 0.05 for significance to confirm whether each candidate was indeed a predictor of the relative abundances of ASVs at the end of the treatment periods. Dietary fiber intake at baseline was negatively associated with the relative abundance of *[Ruminococcus] torques* (q = 0.03) at EndRS2 and negatively associated with the relative abundance of *Oscillospira* at EndRS4 (q = 0.002) (Figure 3). We identified several other ASVs for which the relative abundances at the end of a treatment could be predicted although the prevalence of these ASVs was less than 20% (Table S6). Of these, only one *AMY1*-related metric was predictive; mean SAA was positively associated with the abundance of *Sutterella* at EndCtl (q = 0.04). Interestingly, the carbohydrate-active enzyme (CAZyme) database contains three *Sutterella spp*., *S. faecalis*, *S. megalosphaeroides*, *S. wadsworthensis*, which possess CAZymes.^51^ If the species we detected has CAZymes, the bacterium could be metabolizing host salivary amylase breakdown products that reach the large intestine.

These plots, generated as MaAsLin2 output, show the MaAsLin2 transformed relative abundances of (a) *[Ruminococcus] torques* at the End of RS2 (*n* = 57) and (b) *Oscillospira* at the End of RS4 versus the energy adjusted baseline dietary fiber intake (*n* = 55). Each plot includes a line fitted to the data with the gray shaded area delineating the 0.95 confidence interval. All other significant ASVs had a prevalence below 20% and are listed in Table S5. All q values are in Table S6.

### Changes in fecal SCFA concentration are predicted by treatment order

**Figure 4.**
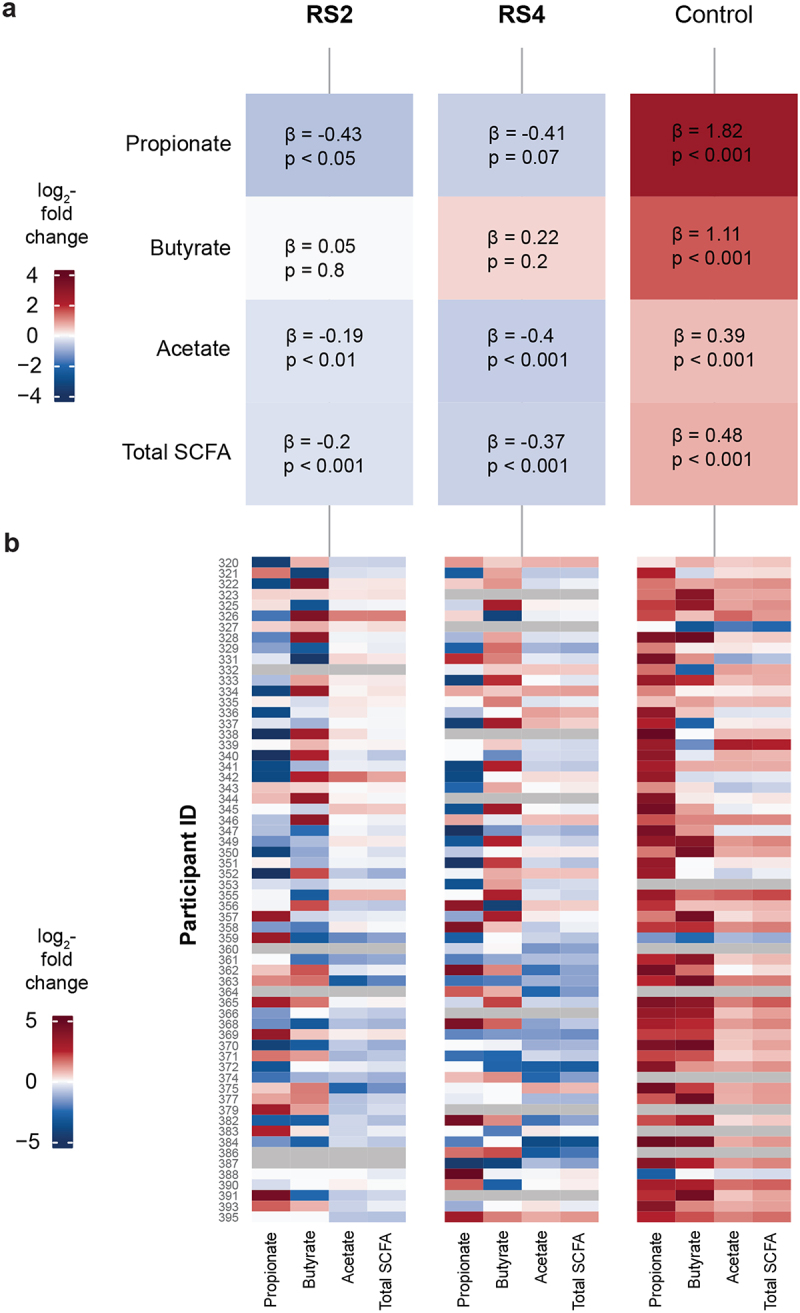
Conserved and variable fecal SCFA response to RS2, RS4, and control treatments.

**Figure 5.**
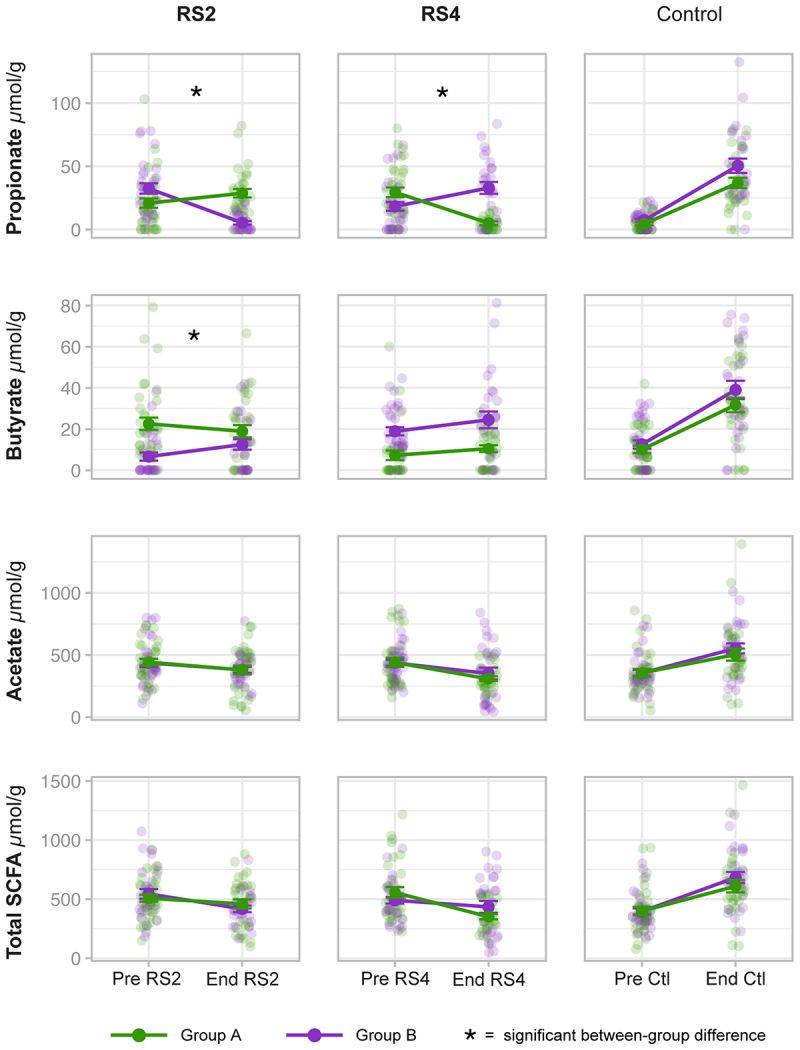
Treatment order predicts propionate response to RS2 and RS4 and butyrate response to RS2.

We found that, at the population level, both RS treatments decreased acetate, propionate, and total SCFA, while Ctl increased acetate, butyrate, propionate, and total SCFA with the strongest effect of the Ctl being on propionate concentration (Figure 4a). Nevertheless, we found interindividual variability in the observed SCFA concentration changes associated with each treatment period (Figure 4b). To determine whether treatment order (Group A versus B) affected changes in SCFA concentrations from Pre to End (time point), we used linear regression models with each SCFA as the response variable and included an interaction term between Group and time point (Figures 5 and S3). We observed a significant interaction between Group and time point when assessing butyrate response to RS2 (*p* = 0.0116); butyrate increased significantly from Pre to End in Group B (*p* = 0.0361) but decreased in Group A (*p* = 0.1357) although not significantly (Figure 5).

(a) This heat map depicts the results of linear mixed models with the time point (Pre and End) of each treatment as a fixed effect, participant as a random effect, and logarithm of the SCFA concentration as the response variable. Coefficient estimates (β) indicate whether the specified SCFA increased or decreased at End compared to Pre for each treatment (RS2: *n* = 57, RS4: *n* = 55, control: *n* = 55). (b) We observed interindividual variability in log_2_ fold changes of fecal SCFA concentrations between Pre and End of each treatment. Missing values are indicated by cells shaded gray (*n* = 62).

These plots show the individual data points as well as the mean and standard error (SE) of SCFA concentrations from Pre to End of each treatment by Group, which indicates treatment order (see Figure 1). Group A is green, and Group B is purple. The data points for each participant at Pre and End are transparent circles, and the means are shown as solid circles. Panels are marked with an asterisk (*) where Group significantly affected the change in concentration as determined by linear regression analysis. Group affected change in propionate during RS2 (*p* = 3.19 × 10^−9^), change in propionate during RS4 (*p* = 1.69 × 10^−7^), and change in butyrate during RS2 (*p* = 0.0116) (RS2: *n* = 57, RS4: *n* = 55, control: *n* = 55).

Group also influenced the propionate response to RS2 (*p* = 3.19 × 10^−9^) and RS4 (*p* = 1.69 × 10^−7^), and interestingly, the Group effect was in opposite directions (Figure 5). Propionate concentration significantly increased from Pre to End in Group A (*p* = 0.0097) but decreased from Pre to End in Group B (*p* < 0.0001) during the RS2 treatment. In contrast, propionate concentration significantly decreased from Pre to End in Group A (*p* < 0.0001) but increased from Pre to End in Group B (*p* = 0.0104) during the RS4 treatment. Closer examination revealed that regardless of the type of RS, propionate increased during the first RS treatment (Treatment 1: PreRS to EndRS), increased again during the Ctl treatment (Treatment 2: PreCtl to EndCtl), but decreased during the third treatment, which was the second RS treatment (Treatment 3: PreRS to EndRS) (Figure S4). Using a linear model that included the baseline and all treatment time points, we found that there was no significant effect of Group on changes in propionate concentration over time when the type of RS is disregarded (*p* = 0.4645; Figure S4). Since both groups responded similarly over time with regard to changes in SCFA concentrations (Figure S4), we created a new MaAsLin2 model that included all ASV data from Group A and Group B together, regardless of the type of RS, to identify the ASVs that changed significantly from Pre to End of the first treatment. We identified 11 ASVs, which included members of the genera *Blautia* and *Coprococcus*, and most of these ASVs were still present in most individuals at PreCtl (Table 2; Figure S5). Thus, these microbes may have contributed to the increase in SCFA concentrations that occurred during the Ctl treatment.

**Table 2.** Differentially abundant ASVs from Pre to End of Treatment 1.

ASV Taxonomy	ASV ID	Coefficient	SE	Non-zero values	p value	q value
*Blautia*	c6c3ab4e828fb40d6e05967b7aac9338	−0.63	0.11	126/126	3.70E–07	0.00012
Clostridiales	0243b68c76049aa1b8c51a20333da950	−0.72	0.17	101/126	0.000083	0.0089
*Parabacteroides distasonis*	878fc53ff6f6333a92673b700951fa1b	0.83	0.21	14/126	0.0001	0.0088
*Dorea*	bd5ef8f372e09bcf6ec35825be4f053c	−0.65	0.15	98/126	0.000077	0.0088
*Ruminococcus*	e367dacc4cf58de7164775e5899d4ab2	1.05	0.27	41/126	0.00023	0.015
*Blautia*	0a68ec564e278b0246c920ad7de41880	−0.47	0.13	115/126	0.00076	0.031
*Coprococcus*	6851a4ee264b56be2fff4686ce269907	−0.49	0.14	106/126	0.00061	0.031
*[Ruminococcus]*	9e3226efaf559eb14a478f880462cb8a	−0.7	0.19	92/126	0.0008	0.031
*Coprococcus*	aac0099991c8518a5268b1a19a84550b	−0.55	0.16	96/126	0.00091	0.031
*Clostridium clostridioforme*	d76d59ec71de0e3b22da0c9cd564d41a	−0.63	0.18	124/126	0.00088	0.031
*Bacteroides*	99deb3c5ecb022ec05609ebd1112a557	−0.64	0.19	108/126	0.0013	0.038

Combining data from participants in both Groups A and B in a MaAsLin2 model, we identified 11 ASVs that were differentially abundant (q < 0.05) from Pre to End of the first treatment. This table shows the ASV ID number, coefficients, standard error (SE), and p and q values. Repeated taxa correspond to ASVs that were assigned the same taxonomy. The non-zero values column shows the fraction of 126 measurements (Pre and End) in the analysis with a relative abundance greater than zero for each of the ASVs (Pre: *n* = 68, End: *n* = 58). Figure S5 shows plots of the relative abundances of these ASVs at Pre and End of Treatment 1 and PreCtl.

### Microbial features predict SCFA responses

**Figure 6.**
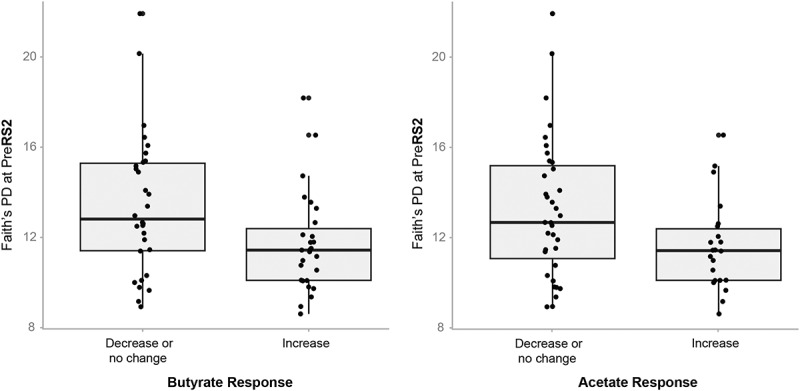
Gut microbiota diversity prior to RS2 treatment predicts increase in butyrate and acetate concentrations in response to RS2 treatment.

To assess whether alpha diversity could predict changes in SCFA concentrations, we defined any increase in SCFA concentration from Pre to End (End – Pre > 0) of the treatment as having a change score of 1, while no change or a decrease in SCFA concentration (End – Pre ≤ 0) was assigned a change score of 0. Alpha diversity, as measured by Faith’s PD, prior to RS2 treatment was a significant predictor of butyrate change score (β = −0.2504, *p* = 0.03) and acetate change score (β = −0.2518, *p* = 0.04) (Figure 6). β coefficient estimates indicate the difference in the log-odds of a response (a change score of 1) between Group A and Group B. In other words, for every unit increase in Faith’s PD at PreRS2, the odds of an increase in butyrate and acetate were each 22% lower. The relationship between lower diversity and acetate and butyrate responses to RS2 may be due to the enrichment of microbes that ferment RS2 or produce these SCFAs at the expense of a decrease in other microbes.

Faith’s PD prior to the consumption of RS2 predicts butyrate response (β = –0.2504, *p* = 0.03) and acetate response (β = –0.25, *p* = 0.04) as determined by logistic regression analysis (*n* = 57). Response was categorized as “Increase” versus “Decrease or no change.”

**Figure 7.**
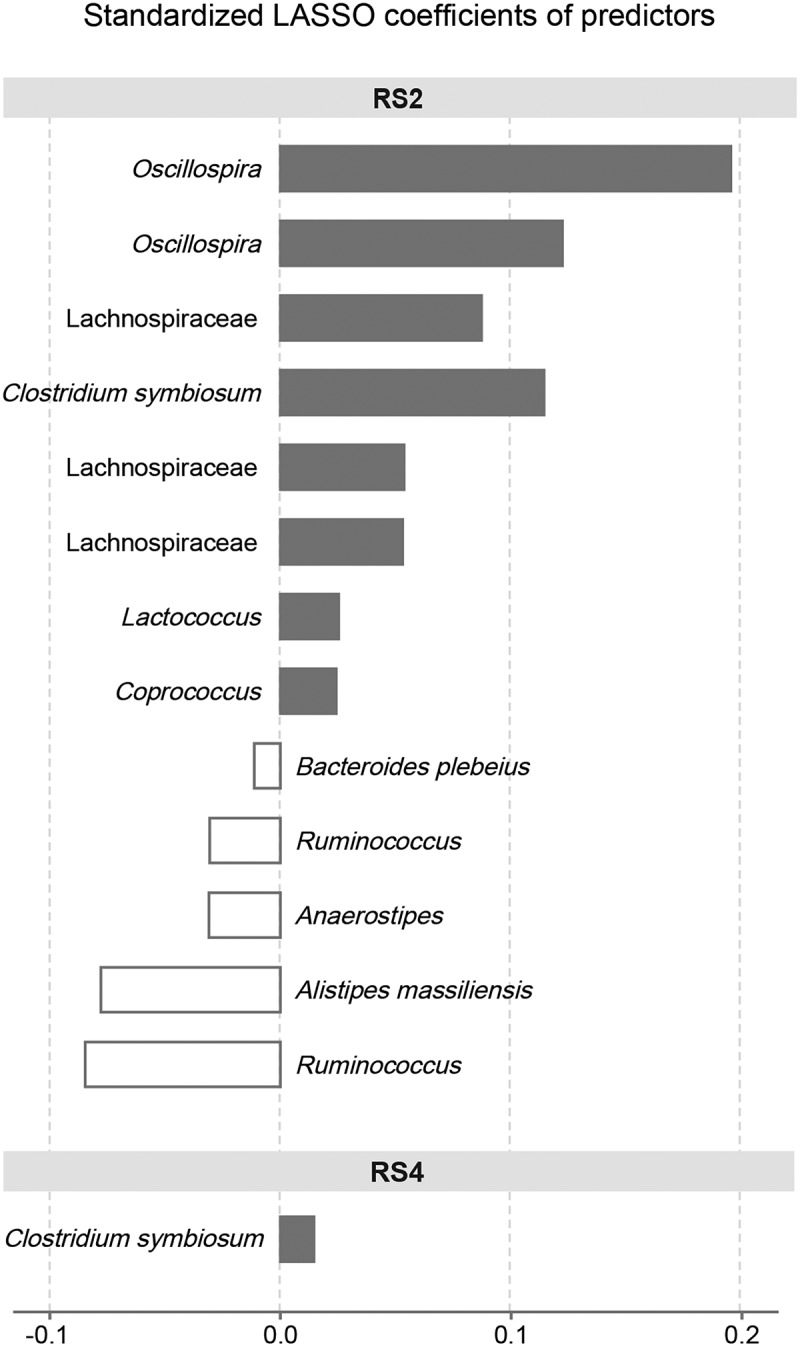
Relative abundances of ASVs at PreRS predict propionate response.

We performed LASSO regression to select the most important variables, including ASVs at Pre, that predict butyrate, propionate, or acetate response to each treatment. We included ASVs at Pre as variables in our models to determine which microbes present in individuals prior to each treatment could be predictive of SCFA response. Additional variables that we included were body fat percentage, sex, treatment order, *AMY1* CN, *AMY1* CN group, mean SAA, and self-reported physical activity (minutes/week of vigorous, moderate, and low-intensity activity) at baseline. When applying LASSO, we were most successful in predicting propionate response (Figure 7; Table S7). We found that 14 variables, treatment order and the relative abundances of 13 ASVs at PreRS2, were predictive of propionate response, defined as the log fold change of propionate between PreRS2 and EndRS2, with R^2^ = 0.64. The ASV with the greatest contribution to the LASSO model for the RS2 treatment was classified as *Oscillospira*. Comparing one participant to another whose Pre relative abundance is 1 standard deviation higher, we expect the log fold change (of End versus Pre) in propionate concentration to increase by nearly 0.2. The ASV with the second greatest contribution for the RS2 treatment was also classified as *Oscillospira*. *Oscillospira* is thought to be associated with SCFA production.^52^ We observed that two variables, treatment order and the relative abundance of *Clostridium symbiosum* at PreRS4, were predictive of propionate response to RS4 (R^2^ = 0.31). Thus, the bacterium *C. symbiosum*, corresponding to the same ASV, was a predictor of propionate response to both RS2 and RS4. This finding suggests that *C. symbiosum* could be involved in the propionate production pathway downstream of substrate degradation. No variables were predictive of changes in acetate or butyrate with an R^2^ > 0.06.

This figure depicts the standardized LASSO coefficients (β*) of the ASVs whose relative abundances at the beginning of treatment predicted propionate response to RS2 and RS4. Propionate response was defined as the log_2_ fold change of propionate between Pre and End of the treatment (Log_2_ [End/Pre]). For each ASV, the standardized beta coefficient tells the expected amount of change in the propionate concentration for each unit of difference (standard deviation from the mean) in relative abundance of the predictive ASV. Bars are shaded when the β coefficient is positive and clear when the β coefficient is negative. Some taxa are repeated because different ASVs were assigned the same taxonomy. In addition to the ASVs shown, treatment order was also identified as an important predictor of propionate response to both RS2 (β = -1.58) and RS4 (β = 1.15) treatments. Treatment order and the ASVs shown together explained 64% of the variability in log_2_ fold change in propionate during RS2 and 31% of the variability in log_2_ fold change in propionate during RS4 (RS2: *n* = 57, RS4: *n* = 55).

## Discussion

Many large, observational studies have linked dietary fiber intake to a decreased incidence of type 2 diabetes and cardiovascular disease.^53–59^ Despite public outreach surrounding its importance, less than 10% of people eat the dietary reference intake for dietary fiber (women: 25 g, men: 38 g).^60^ Many of the health-related benefits of dietary fiber are mediated by microbial metabolites such as SCFAs.^61^ The consumption of RS, a form of dietary fiber, has previously been associated with increases in fecal SCFA concentrations, improvements in glucose metabolism, and decreases in serum cholesterol. However, reports of beneficial health effects have been conflicting, and interindividual variability in microbiota response has frequently been observed.^7,9,10,48–50,62^ Microbial community membership differs between individuals, and different gut microbes produce different metabolites. These differences are thought to be responsible, at least in part, for variability in the effects of dietary fiber sources on gut microbiome composition and metabolic health parameters between individuals. An area of active study is how to develop precision nutrition strategies to prescribe the best source of dietary fiber for each individual to prevent chronic disease.

Our most striking results were that (i) treatment order (the order in which participants received RS2 and RS4) was a significant predictor of the change in propionate concentration from Pre to End of both RS2 and RS4 supplementation and (ii) the control cracker, containing the digestible starch, caused greater increases in SCFA concentrations than the RS crackers. A previous group conducted *in* *vitro* experiments in which they inoculated a Simulator of the Human Intestinal Microbial Ecosystem (SHIME) with human stool.^63^ They observed enhanced SCFA concentrations following a week-long administration of the same AMIOCA™ TF corn starch (Ingredion) that we used. Thus, SCFAs can be produced by human gut microbiota using this digestible starch substrate. In another *in* *vitro* study using human fecal suspensions, the authors observed a greater increase in butyrate production when corn starch was the substrate than when a cabbage-fiber mixture was used.^64^

There are also human feeding studies that support our finding that the control crackers caused an increase in fecal SCFA concentration. In a previous study, human participants were fed a diet rich in digestible starch for two 4-week periods.^65^ The participants were given acarbose during one of the two periods and placebo in the other. Acarbose inhibits starch digestion in the small intestine. Fecal SCFA (acetate, n-butyrate, and total SCFAs) concentrations were significantly higher during the period that included acarbose. Thus, when the acarbose treatment allowed the digestible starch to reach the large intestine, the result was an increase in fecal concentration of SCFAs. In another study, human participants’ diets were supplemented with AMIOCA™ TF (Ingredion), which was designated as the corn starch control.^12^ At a dose of 50 g per day, researchers observed an increase in fecal concentrations of total SCFAs, acetate, propionate, and butyrate. Interestingly, in our study, MaAsLin2 did not identify any ASVs with significant differential abundance between PreCtl and EndCtl at q < 0.05 suggesting that there were no changes in the community at the ASV level during the Ctl treatment. Nevertheless, shotgun metagenomics data could reveal changes in the relative abundances of taxa identified at a higher resolution (at the species or strain level) or changes in the relative abundances of gene functions such as carbohydrate-active enzymes and genes involved in pathways that generate SCFAs during the Ctl treatment. Additionally, metatranscriptomics may be informative because the transcriptional activity of the microbial communities may have changed.

In all the participants in our study, the gut microbiota was exposed to one of two different types of RS prior to receiving the AMIOCA™ TF, and this exposure during the first treatment may have increased the abundance of microbes involved in SCFA production, an effect that may have lingered beyond the 5-d washout. This suggests that the washout periods were insufficient or that the control cracker had a lingering impact on SCFA concentrations. When we included the relative abundances of ASVs at Pre and End of the first treatment from both groups A and B in a MaAsLin2 model, we identified 11 ASVs which significantly changed in relative abundance between Pre and End, and the assigned taxonomy included *Bacteroides*, *Ruminococcus*, and *Coprococcus*, genera with species implicated in SCFA production and/or carbohydrate degradation.^66,67^ All 11 ASVs were still present in at least a subset of participants at PreCtl, the beginning of the control treatment, during which SCFA concentrations increased. Future *in* *vitro* experiments could reveal whether the presence of these taxa is instrumental in altering SCFA concentrations when RS or digestible starch substrate is present.

Propionate concentration decreased significantly during the third treatment, when Group B consumed RS2 and Group A consumed RS4. SCFA production involves consortia of microbes acting in a multi-step process in which some microbes degrade the carbohydrate substrate, while others cross-feed on intermediary metabolites to produce acetate, butyrate, and propionate.^68,69^ One explanation could be that the first treatment caused changes in the community’s functional capacity, which enhanced RS2 degradation in Group A and RS4 degradation in Group B. Then, the specialization of the gut microbiota for degradation of RS2 or RS4 remained at the start of the third treatment, when the alternative RS was consumed by each participant. Another possibility is that over time the control cracker depleted some of the microbes in the consortia required to generate propionate from RS, so much so that the subsequent 5-d washout period was not long enough for the microbes to rebound. Since we could not find evidence supporting these notions based on analyses of 16S rRNA sequence data, shotgun metagenomic sequencing of community DNA or RNA may be required to resolve the species and gene functions underlying the community dynamics that caused the observed effects.

We also found that lower alpha diversity prior to RS2 consumption was associated with higher odds of acetate and butyrate responses to RS2. The relationship between lower diversity and acetate and butyrate responses to RS2 may be due to the enrichment of microbes that ferment RS2 or produce SCFAs at the expense of a deficit of many other microbes. The fact that LASSO did not identify ASVs that were predictive of changes in butyrate or acetate could be due to interindividual variability in the species that are involved in butyrate and acetate production in the context of our study. We also tried several other previously published statistical techniques to identify predictors of SCFA response, but these analyses were uninformative, possibly due to a lack of sufficient power. Future studies should be powered accordingly.

Our study does have some limitations. The treatments could have caused the participants to adjust their dietary intake, e.g. the study crackers could have replaced another component of their usual diet or may have increased their overall caloric intake, which could have modified the effects attributed to the treatments. This is an inherent challenge of any dietary intervention study in which participants can consume their usual diets *ad libitum*. Nevertheless, the advantages of our approach were that participants were not asked to abandon their preferred habitual intake and the RS was provided in a purified form as opposed to being added in the form of RS-rich whole foods, which assured uniformity in supplementation of the RS types.

Additionally, as in the vast majority of studies of the human gut microbiome, our findings are subject to the caveats of using stool samples to represent changes in the composition and function of colonic microbes. For example, we were limited to capturing the 5–10% of SCFAs that are excreted in stool.^70^ Stool SCFA content is typically used as a proxy for the amount of SCFAs produced by colonic microbes in microbiome studies because more accurate procedures would be impractical for human participant studies. Unfortunately, this proxy is not equal to the amount of SCFAs produced by the colonic microbes because most SCFAs are rapidly absorbed by colonocytes. Thus, the changes in SCFA concentrations that we observed could be due to changes in the absorption rate in the gut lumen as opposed to changes in production. Additionally, microbiome composition differs somewhat between the colon and stool.^71^

Another limitation of our study is that the 5-d washout periods may have been insufficient. We believed that 5 d would be sufficient based on two previous, highly cited dietary intervention studies that assessed day-to-day changes in microbiome composition in response to changes in diet.^72,73^ Both studies reported that changes in diet caused community differences within 1 d. In fact, one of the studies, David et al.,^72^ observed that, “… subjects’ gut microbiota reverted to their original structure 2 d after the animal-based diet ended.” Thus, we reasoned that the microbial communities should return to their native composition after 5 d of the participants’ regular diet without the crackers. In case of a carryover effect, we had all participants consume the control cracker during the second treatment because most previous work suggests that digestible starch has a limited effect on the gut microbiome.

We used MaAsLin2 models to assess whether microbiome composition differed between the time points at the beginning of each of the RS treatments, Treatment 1 and Treatment 3. We determined that there were no statistically significant ASVs with differential abundance between Pre of Treatment 1 and Pre of Treatment 3 (q < 0.05). Thus, at the ASV level, community composition was not significantly different at the beginning of each of the RS treatments. When we used MaAsLin2 to compare the time points, Pre of Treatment 1 and Pre of Treatment 2 (Ctl), we identified a single ASV (1 of the 333 ASVs tested in the model) assigned to the genus *Adlercreutzia* in the phylum Actinobacteria that was differentially abundant (q < 0.05). *Adlercreutzia* is a known gut commensal that includes species reported to metabolize daidzin and resveratrol.^74^ Determining whether the ASV, corresponding to *Adlercreutzia*, played a role in the increased fecal SCFA concentrations observed during the Ctl treatment would require additional experiments.

Individualized gut microbial responses have the potential to influence the metabolic effects of the consumption of dietary fibers such as RS. Of note, RS is found in many commonly consumed foods, including breads, cereals, bananas, grains, pasta, rice, legumes, and potatoes.^75^ In this study, we identified several factors that predict the variable response of gut microbiota to RS2 and RS4. We observed that alpha diversity and the relative abundances of select ASVs prior to RS supplementation predicted changes in fecal SCFA concentrations following RS supplementation. SCFAs are involved in numerous signaling pathways that benefit metabolic health.^5,61,76^ Thus, we expect that people who have increased fecal concentrations of SCFAs in response to RS consumption are more likely to have improvements in glucose and lipid metabolism. If this is the case, assessing predictive microbial features prior to suggesting dietary changes could help determine whether consumption of foods containing RS is likely to be effective. Future work is required to determine whether non-responders to RS are more likely to respond to some other form of dietary fiber, e.g. nonstarch polysaccharides. Our findings also suggest that brief intervals of digestible carbohydrate supplementation may enhance SCFA production although the short- and long-term impact on metabolic health is currently unknown.

## Supplementary Material

Supplemental Material

## Data Availability

The raw fastq sequences generated from the 16S rRNA amplicon sequencing are available in the National Center for Biotechnology Information Sequence Read Archive via the project number PRJNA944997.
