# Supplementary material for: Gut microbial features and dietary fiber intake predict gut microbiota response to resistant starch supplementation
Source: Gut Microbes. 2024 Jun 24;16(1):2367301. doi: 10.1080/19490976.2024.2367301 (PMC11197919; doi:10.1080/19490976.2024.2367301)
Supplement: Supplemental Material [file KGMI_A_2367301_SM8723.zip › Supplemental tables 20240417.docx]

**Supplemental materials**

Gut microbial features and dietary fiber intake predict gut microbiota response to resistant starch supplementation

**Table S1.** **Inclusion and exclusion criteria.**

This table provides the inclusion and exclusion criteria for study participants.

| **Inclusion criteria** | **Exclusion criteria** |
| --- | --- |
| Aged 18 years or above | History of diabetes, prediabetes or impaired glucose tolerance |
| Willing to have height, weight, waist, and hip circumference measured | An existing, UNTREATED, thyroid condition |
| Willing to provide 4 saliva samples, 12 stool samples over ~7 weeks of the study duration | Use of systemic antibiotics (intravenous injection, intramuscular, or oral) within the last 6 months |
| Stable weight (±5 pounds) for the last three months | An acute disease at the time of enrollment (Acute disease is defined as the presence of a moderate or severe illness with or without fever). |
|  | A chronic, clinically significant (unresolved, requiring ongoing medical management or medication) pulmonary, cardiovascular, gastrointestinal, hepatic, or renal functional abnormality |
|  | A history of active uncontrolled gastrointestinal disorders or diseases including  A. Inflammatory bowel disease (IBD)  i.) Ulcerative colitis (mild-moderate-severe)  ii.) Crohn’s disease (mild- moderate-severe)  B. Indeterminate colitis  C. Irritable bowel syndrome (IBS) (moderate-severe)  D. Persistent, infectious gastroenteritis, colitis or gastritis, persistent or chronic  diarrhea of unknown etiology  E. Clostridium difficile infection (recurrent)  F. Chronic constipation |
|  | Bariatric surgery |
|  | An unstable dietary history as defined by major changes in diet during the previous month. For example, elimination or significantly increased intake of a major food group in the diet. |
|  | A recent history of chronic alcohol consumption defined as more than 5 drinks (or servings) of alcohol per day. |
|  | Female participants: Pregnancy and/or lactation |

**Table S2a.** **Study cracker formulations.**

RS2 crackers contained HI-MAIZE® 260 starch (56% RS), RS4 crackers contained VERSAFIBE^TM^ 1490 starch (85% RS), and Control crackers contained AMIOCA™ TF starch (100% digestible starch) (Ingredion Inc., Bridgewater, N.J., USA).

| **Ingredient** | **Control** | **RS2** | **RS4** |
| --- | --- | --- | --- |
| Bread flour | 49.85% | 29.40% | 45.27% |
| Amioca TF starch | 24.72% | - | - |
| HiMaize 260 | - | 44.78% | - |
| VersaFibe 1490 | - | - | 29.95% |
| Unsalted Butter | 17.89% | 17.59% | 18.05% |
| Sugar | 2.41% | 2.36% | 2.44% |
| Salt | 1.15% | 1.12% | 1.15% |
| Water | 3.99% | 4.76% | 3.14% |
| TOTAL | 100.0% | 100.0% | 100.0% |

**Table S2b.** **Macronutrient and calorie content of the study crackers.**

From day 4 to day 10 of the treatment, participants received the full dose of crackers, 120g. The macronutrient content of each cracker is shown in grams. Our top priority was to match the crackers for total carbohydrate content. As a result, the calories in the control cracker are higher than in RS2 and RS4.

**Control**

| **Per 100g cracker** | **Amioca TF** | **Butter+Sugar+salt** | **Bread ﬂour** | **Total** |
| --- | --- | --- | --- | --- |
| kcal | 88.50 | 137 | 182.78 | 408.28 |
| Carbs (g) | 22.075 | 2.42 | 38.22 | 62.71 |
| Fat (g) | 0 | 14.5 | 0 | 14.5 |
| Protein (g) | 0.025 | 0.14 | 6.64 | 6.8 |

**RS2**

| **Per 100g cracker** | **Hi-maize** | **Butter+Sugar+salt** | **Bread ﬂour** | **Total** |
| --- | --- | --- | --- | --- |
| kcal | 77.02 | 135 | 107.8 | 319.82 |
| Carbs (g) | 39.68 | 2.37 | 22.54 | 64.59 |
| Fat (g) | 0.36 | 14.26 | 0 | 14.62 |
| Protein (g) | 0.27 | 0.14 | 3.92 | 4.33 |

**RS4**

| **Per 100g cracker** | **Versaﬁbe** | **Butter+Sugar+salt** | **Bread flour** | **Total** |
| --- | --- | --- | --- | --- |
| kcal | 14.08 | 138 | 165.99 | 318.06 |
| Carbs (g) | 25.76 | 2.45 | 34.71 | 62.91 |
| Fat (g) | 0 | 14.64 | 0 | 14.64 |
| Protein (g) | 0 | 0.14 | 6 | 6.18 |

**Table S3.** **RS and total dietary fiber content in study crackers.**

Content of resistant starch and total dietary fiber in the study crackers as analyzed by Medallion Laboratories (Minneapolis, MN) using the methods AOAC 2002.02, which directly measures RS in the form of RS2 but not RS4, and AOAC 991.43 which measures total dietary fiber and detects both RS2 and RS4 [(1)](https://www.zotero.org/google-docs/?2TTmS5). Since AOAC 2002.02 does not accurately detect RS4, this measurement is not indicative of the amount of RS in the RS4 cracker [(2)](https://www.zotero.org/google-docs/?BISGbJ). Since AOAC 991.43 does detect RS4, by deduction, the AOAC 991.43 measurement is indicating the approximate amount of RS4 (less the 1.92g of total dietary fiber from the other ingredients, which are also in the control cracker) [(3)](https://www.zotero.org/google-docs/?pxsRn0). Since AOAC 991.43 is not a direct measurement of RS4, it is possible that the final proportion of RS4 in the total dietary fiber content measured by the AOAC 991.43 method may have been reduced by the baking process, as it was for the RS2 cracker. Amounts in the table are per 120 g of crackers, which was the daily portion of study crackers per each participant after the gradual dose escalation period.

|  | **Control Cracker** | **RS2 Cracker** | **RS4 Cracker** |
| --- | --- | --- | --- |
| AOAC 2002.02 | < 2.4 g | 21.27 g | <2.4 g |
| AOAC 991.43 | 1.92 g | 35.64 g | 36 g |

[1. McCleary BV, Collaborators: Total Dietary Fiber (CODEX Definition) in Foods and Food Ingredients by a Rapid Enzymatic-Gravimetric Method and Liquid Chromatography: Collaborative Study, First Action 2017.16. Journal of AOAC INTERNATIONAL 2019;102:196–207.](https://www.zotero.org/google-docs/?q1ohmQ)

[2. Coulon DB, Page R, Raggio AM, Guice J, Marx B, Gourineni V, Stewart ML, Keenan MJ. Novel Resistant Starch Type 4 Products of Different Starch Origins, Production Methods, and Amounts Are Not Equally Fermented when Fed to Sprague-Dawley Rats. Mol Nutr Food Res 2020;64:e1900901.](https://www.zotero.org/google-docs/?q1ohmQ)

[3. Fiber Methods: 32-01.01 Guidelines on Method Selection for Dietary Fiber Analysis [Internet]. AACC Approved Methods of Analysis, 11th Edition. 2023 [cited 2024 Apr 14]. Available from: https://www.cerealsgrains.org/resources/Methods/Pages/32Fiber.aspx](https://www.zotero.org/google-docs/?q1ohmQ)

**Table S4. Significantly different ASVs between Pre and End of each treatment period as determined by MaAsLin2.**

This table shows 𝛽 coefficients for the ASVs that were significantly different in relative abundance (q<0.05) from Pre to End of each treatment. SE: standard error.

| **Treatment** | **ASV** | **Coefficient** | **SE** | ***p* value** | **q value** |
| --- | --- | --- | --- | --- | --- |
| RS2 | *Blautia* | -0.88 | 0.10 | <0.001 | <0.001 |
|  | *Coprococcus* | -0.68 | 0.11 | <0.001 | <0.001 |
|  | *Adlercreutzia* | 0.66 | 0.13 | <0.001 | <0.001 |
|  | *Dorea* | -0.79 | 0.15 | <0.001 | <0.001 |
|  | Erysipelotrichaceae | -1.3 | 0.24 | <0.001 | <0.001 |
|  | *Blautia obeum* | -0.72 | 0.14 | <0.001 | <0.001 |
|  | *Dorea formicigenerans* | -0.56 | 0.12 | <0.001 | <0.001 |
|  | Lachnospiraceae | -0.73 | 0.15 | <0.001 | <0.001 |
|  | *[Ruminococcus] torques* | -1.1 | 0.23 | <0.001 | <0.001 |
|  | *Clostridium clostridioforme* | -0.72 | 0.16 | <0.001 | 0.001 |
|  | *Coprococcus* | -0.54 | 0.12 | <0.001 | 0.002 |
|  | *Clostridium* | -1.0 | 0.24 | <0.001 | 0.002 |
|  | *Blautia* | -0.55 | 0.14 | <0.001 | 0.003 |
|  | *Parabacteroides distasonis* | 0.60 | 0.15 | <0.001 | 0.004 |
|  | *Oscillospira* | -0.65 | 0.17 | <0.001 | 0.008 |
|  | Clostridiales | -0.56 | 0.15 | <0.001 | 0.01 |
|  | *Oscillospira* | -0.73 | 0.21 | <0.001 | 0.01 |
|  | Ruminococcaceae | 0.71 | 0.20 | <0.001 | 0.02 |
|  | Lachnospiraceae | -0.88 | 0.26 | 0.001 | 0.02 |
|  | *[Ruminococcus]* | -0.71 | 0.21 | 0.001 | 0.02 |
|  | *Blautia* | -0.42 | 0.13 | 0.002 | 0.02 |
|  | *Coprococcus* | -0.52 | 0.16 | 0.0021 | 0.03 |
|  | *Anaerostipes* | -0.37 | 0.12 | 0.003 | 0.04 |
|  | *Lachnospira* | -0.65 | 0.21 | 0.003 | 0.04 |
|  | *Blautia* | 0.79 | 0.26 | 0.003 | 0.04 |
|  | Lachnospiraceae | -0.32 | 0.10 | 0.003 | 0.04 |
|  | *[Ruminococcus] gnavus* | -0.83 | 0.27 | 0.003 | 0.04 |
|  | Lachnospiraceae | -0.66 | 0.22 | 0.004 | 0.04 |
|  | *Blautia producta* | -0.52 | 0.18 | 0.004 | 0.04 |
|  | Lachnospiraceae | -0.30 | 0.10 | 0.004 | 0.04 |
|  | *Ruminococcus bromii* | 1.1 | 0.34 | 0.004 | 0.04 |
|  | Ruminococcaceae | 0.38 | 0.13 | 0.004 | 0.04 |
|  | *Faecalibacterium prausnitzii* | 0.46 | 0.15 | 0.004 | 0.04 |
|  | *Bacteroides* | -0.50 | 0.17 | 0.005 | 0.04 |
| RS4 | *Parabacteroides distasonis* | 0.95 | 0.18 | <0.001 | 0.0001 |
|  | *Ruminococcus* | 1.28 | 0.23 | <0.001 | 0.0001 |
|  | *Blautia* | -0.66 | 0.12 | <0.001 | 0.0001 |
|  | Lachnospiraceae | 1.0 | 0.23 | <0.001 | 0.004 |
|  | *Parabacteroides distasonis* | 0.77 | 0.19 | <0.001 | 0.005 |
|  | *Parabacteroides distasonis* | 0.85 | 0.21 | <0.001 | 0.005 |
|  | Lachnospiraceae | -0.66 | 0.16 | <0.001 | 0.006 |
|  | Lachnospiraceae | 1.5 | 0.37 | <0.001 | 0.007 |
|  | Clostridiales | -0.65 | 0.16 | <0.001 | 0.007 |
|  | *Clostridium* | -0.96 | 0.25 | <0.001 | 0.01 |
|  | Lachnospiraceae | 0.89 | 0.24 | <0.001 | 0.01 |
|  | *Dorea* | -0.54 | 0.14 | <0.001 | 0.01 |
|  | *Blautia* | -0.38 | 0.11 | <0.001 | 0.02 |
|  | *Bacteroides ovatus* | 1.0 | 0.29 | 0.001 | 0.02 |
|  | *Bacteroides ovatus* | 0.75 | 0.22 | 0.001 | 0.02 |
|  | *Bacteroides* | -0.46 | 0.13 | 0.001 | 0.02 |
|  | *[Ruminococcus] torques* | -0.84 | 0.25 | 0.001 | 0.03 |
|  | Lachnospiraceae | -0.67 | 0.20 | 0.001 | 0.03 |
|  | *Dorea* | -0.60 | 0.19 | 0.002 | 0.04 |
|  | *Dorea formicigenerans* | -0.31 | 0.10 | 0.003 | 0.04 |
|  | Lachnospiraceae | -0.64 | 0.21 | 0.003 | 0.046 |
|  | Lachnospiraceae | -0.44 | 0.14 | 0.003 | 0.046 |
|  | Clostridiales | 1.1 | 0.37 | 0.003 | 0.046 |
|  | *Ruminococcus* | 0.64 | 0.21 | 0.003 | 0.046 |

**Table S5. ASVs with q<0.25 interaction terms between Pre and End of each treatment period as determined by MaAsLin2.**

ASVs in which we observed interaction effects (q<0.25) between candidate predictors (energy-adjusted dietary fiber intake during treatment, energy-adjusted dietary fiber intake during baseline, treatment order, *AMY1* Group, and mean SAA) and time point type (Pre vs End of treatment). Since this was a screening step we raised our threshold of significance to q<0.25. SE: Standard Error.

| **Interaction tested** | **Treatment** | **ASV** | **Coefficient** | **SE** | ***p* value** | ***q* value** |
| --- | --- | --- | --- | --- | --- | --- |
| Fiber intake during each treatment period * time point (Pre vs. End) | RS2 | *Oscillospira* | -1.58 | 0.44 | <0.001 | 0.14 |
|  | RS4 | ⸻ | ⸻ | ⸻ | ⸻ |  |
|  | Control | ⸻ | ⸻ | ⸻ | ⸻ |  |
| Fiber intake at baseline * time point (Pre vs. End) | RS2 | Ruminococcaceae | 0.74 | 0.23 | 0.002 | 0.17 |
|  | RS4 | Clostridiales | -0.68 | 0.20 | 0.002 | 0.15 |
|  | Control | *Veillonella dispar* | -1.6 | 0.463 | <0.001 | 0.13 |
| Treatment order (Group A vs Group B) * time point (Pre vs End) | RS2 | *Blautia producta* | -0.22 | 0.071 | 0.004 | 0.21 |
|  |  | Clostridiaceae | -0.77 | 0.26 | 0.005 | 0.21 |
|  |  | Rikenellaceae | -0.36 | 0.13 | 0.006 | 0.21 |
|  |  | Ruminococcaceae | -0.66 | 0.23 | 0.005 | 0.21 |
|  | RS4 | *[Ruminococcus] gnavus* | 0.30 | 0.089 | 0.001 | 0.12 |
|  |  | *Coprococcus* | 0.32 | 0.11 | 0.005 | 0.24 |
|  | Control | ⸻ | ⸻ | ⸻ | ⸻ | ⸻ |
| *AMY1* Group (High vs Low)* time point (Pre vs End) | RS2 | *[Ruminococcus]* | 0.49 | 0.13 | <0.001 | 0.08 |
|  |  | *Erysipelotrichaceae Dielma* | 0.88 | 0.25 | 0.001 | 0.14 |
|  | RS4 | ⸻ | ⸻ | ⸻ | ⸻ | ⸻ |
|  | Control | ⸻ | ⸻ | ⸻ | ⸻ | ⸻ |
| Mean salivary amylase activity * time point (Pre vs. End) | RS2 | *Sutterella* | -0.57 | 0.16 | <0.001 | 0.13 |
|  |  | *Slackia* | -0.28 | 0.086 | 0.002 | 0.15 |
|  |  | *Methanobrevibacter* | 0.22 | 0.073 | 0.003 | 0.24 |
|  | RS4 | *Lachnospiraceae* | -0.58 | 0.15 | <0.001 | 0.19 |
|  | Control | *Sutterella* | 0.62 | 0.14 | <0.001 | 0.045 |

**Table S6. Candidates that predict the relative abundance of ASVs at the End of treatment.**

Shown here are the candidates that significantly predict the relative abundance of ASVs at the End of treatment (q<0.05). The 𝛽 column represents the change in relative abundance for the specified ASV for each unit of change in the specified candidate predictor variable. SE: standard error. In addition, the non-zero values column shows the fraction of samples (Pre and End) in the analysis with a relative abundance greater than zero for each of the ASVs.

| **Treatment** | **Candidate Predictor** | **ASV Taxonomy** | **ASV ID** | **𝛽** | **SE** | **Non-zero values** | **p value** | **q value** |
| --- | --- | --- | --- | --- | --- | --- | --- | --- |
| RS2 | Dietary fiber intake during Baseline | *[Ruminococcus] torques* | X08bd43071a06abf56df235fca6655bea | -1.37 | 0.33 | 34/57 | 0.0001 | 0.03 |
|  |  | *Dialister* | a1580b985a39db6e693626a2e4f28d6d | 0.49 | 0.12 | 6/57 | 0.0002 | 0.03 |
|  | Dietary fiber intake during RS2 | *Coprococcus* | cb89e1b7ff82a658c8def6e75f5c14bc | 0.44 | 0.1 | 10/55 | 0.0001 | 0.04 |
| RS4 | Dietary fiber intake during Baseline | *Oscillospira* | X77c2dc197e6b3dbebc4ee240c6a1c559 | -1.4 | 0.27 | 39/55 | 6.45x10-6 | 0.002 |
| Ctl | Mean SAA | *Sutterella* | dbcdaca3ee9e9fe75d8829e87b8551dc | 1.3 | 0.31 | 10/55 | 9.99x10-5 | 0.04 |

**Table S7. Treatment order and ASVs at Pre RS are predictive of propionate response to RS.**

This table shows the contributions of independent variables included in LASSO models to predict the log fold change of propionate concentrations, as represented by their 𝛽 coefficient values. The relative abundances of the ASVs at Pre of the specified treatment period were used in the models. Repeated taxa within the same treatment indicate the same taxonomic assignment for different ASVs. Treatment order and the ASVs together explained 64% of the variability in log fold change in propionate during RS2 and 31% of the variability in log fold change in propionate during RS4.

| Treatment | Predictor | ASV ID | LASSO coefficient | Standardized  LASSO  coefficient |
| --- | --- | --- | --- | --- |
| RS2 | *Oscillospira* | 1a7a9d14747af9f14fcdabdef2549a77 | 2428.8 | 0.1952 |
|  | *Oscillospira* | 7e7d68f511a7019ad4a8d6b9b8138e34 | 381.87 | 0.1222 |
|  | *Clostridium symbiosum* | 67e8859e108281ee7971084bfa759522 | 272.62 | 0.1142 |
|  | Lachnospiraceae | b1d8e09561b600103cc3d8b388c02478 | 75.5 | 0.05359 |
|  | Lachnospiraceae | 19715b1593c19aa6d35a4d1e3cd09374 | 69.35 | 0.08716 |
|  | *Lactococcus* | d114fb4c335125128be28401522dd41a | 33.15 | 0.02525 |
|  | Lachnospiraceae | 96040114b5274bdb987f28c63b6b6b87 | 31.31 | 0.05297 |
|  | *Coprococcus* | 6851a4ee264b56be2fff4686ce269907 | 3.54 | 0.02418 |
|  | *Bacteroides plebeius* | 47c305878bfc73bfeda34b2ae1a82ee1 | -0.39 | -0.0113 |
|  | *Ruminococcus* | d9eea834805833ac21a86fdabedd8556 | -7.16 | -0.08455 |
|  | *Anaerostipes* | 421cbd1d71d34704c6d53377261e213c | -19.98 | -0.03093 |
|  | *Alistipes massiliensis* | 9d583502f595e3a521989b645d50e327 | -23.35 | -0.07773 |
|  | *Ruminococcus* | d4454a4ebd63a8e375dadd2632588f59 | -109.28 | -0.03061 |
|  | Treatment Order | NA | -1.58 | NA |
| RS4 | *Clostridium symbiosum* | 67e8859e108281ee7971084bfa759522 | 31.69 | 0.01454 |
|  | Treatment Order | NA | 1.15 | NA |
